# EIF5A2 specifically regulates the transcription of aging-related genes in human neuroblastoma cells

**DOI:** 10.1186/s12877-023-03793-6

**Published:** 2023-02-07

**Authors:** Yuwei Liu, Li Peng, Jing Chen, Ling Chen, Ying Wu, Mengxin Cheng, Min Chen, Xujun Ye, Yalei Jin

**Affiliations:** 1 Department of Internal Medicine and Geriatrics, Zhongnan Hospital of Wuhan University, Wuhan University, Wuhan, Hubei China; 2 Department of General Practice, Zhongnan Hospital of Wuhan University, Wuhan University, Wuhan, Hubei China; 3 Department of Cardiology, Zhongnan Hospital of Wuhan University, Wuhan University, Wuhan, Hubei China

**Keywords:** EIF5A2, RNA-seq, Transcription, Aging, Unfolded protein response

## Abstract

**Background:**

Post-transcriptional regulation plays a critical role in controlling biological processes such as aging. Previous studies have shown that eukaryotic initiation factor 5A (EIF5A) might play a crucial role in aging. It is unknown whether EIF5A2, a second isoform of EIF5A, could impact aging through post-transcriptional regulation.

**Methods:**

In the present study, EIF5A2 overexpression (EIF5A2-OE) was induced in SH-SY5Y cells. RNA-seq, bioinformatics analysis and RT-qPCR validation experiments were then performed to explore the molecular mechanism of EIF5A2-mediated transcriptional regulation. Cell viability, proportion of senescent cells and the cell cycle were respectively determined by Cell Counting Kit-8, SA-β‑galactosidase and flow cytometry to evaluate the cell senescence.

**Results:**

A total of 190 downregulated and 126 upregulated genes related to EIF5A2-OE were identified. Genes closely related to cellular aging processes such as unfolded protein response (UPR), cell adhesion and calcium signaling pathway were under global transcriptional regulation. Moreover, EIF5A2-OE promoted the viability of SH-SY5Y cells and reduced cell senescence in vitro. Among 30 genes with the most significant expression differences in EIF5A2-OE cells, we identified eight genes, including *ASNS*, *ATF3*, *ATF4*, *CEBPB*, *DDIT3*, *HERPUD1*, *HSPA5* and *XBP1,* enriched in the UPR. Through EIF5A2-tanscription factors (TFs)-targets regulation network in EIF5A2-OE cells, we found three TFs, BHLHE40, RHOXF1 and TBX20, that targeted at these eight UPR-related genes. Verification test via the published database of human glial cell tissue showed only BHLHE40 and RHOXF1 were significantly associated with EIF5A2.

**Conclusions:**

Our findings suggest that EIF5A2 may alleviate cell senescence in vitro and mediate UPR-related genes via specific TFs. Thus, EIF5A2 could function as a regulator of aging via the regulation of transcription, which greatly expands the current understanding of the mechanisms of EIF5A2**-**mediated gene regulation.

**Supplementary Information:**

The online version contains supplementary material available at 10.1186/s12877-023-03793-6.

## Introduction

It has been fully elucidated that aging is a major risk factor for neurodegenerative diseases, carcinoma, and many other chronic diseases [1]. Although there is no single molecular mechanism that can explain the functional decline in so many systems in the elderly, one dominant theory that has been put forward is that molecular damages, genome injuries and instability, accumulates over time, leading to phenotypic changes in organisms [2]. Even if chemically more vulnerable than DNA, RNAs are usually rapidly turned over. Thus, the consequences of damaged RNA molecules are usually restricted.

The regulation of gene expression tends to occur in the transcriptional stage. However, post-transcriptional regulation plays a critical role in controlling biological processes as well. Post-transcriptional regulation has been reported to participate in many gene expression processes such as apoptosis, immune response, inflammation, the cell cycle, and oncogenesis [3]. RNA binding proteins participate in many aspects of post-transcriptional regulation, including pre-mRNA splicing, alternative splicing, and regulation of mRNA stability, transport, and translation [4].

Eukaryotic initiation factor 5A (EIF5A) is known to act as a translation initiation factor specific for a small number of mRNAs and is highly conserved throughout evolution [5]. EIF5A was first isolated from rabbit reticulocyte lysate ribosomes [6], but later studies suggested that it plays a more ubiquitous role in cells, including cellular proliferation and apoptosis [7], and nucleocytoplasmic transport [8]. Moreover, it is reported to be involved in various aspects of RNA metabolism, including mRNA degradation and turnover [9]. Several studies have implicated EIF5A in aging. Schrader et al. reported that EIF5A mediates cell viability and senescence through its effects on the stability of certain mRNAs [10]. Decreased expression of EIF5A leads to mitochondrial fission and excessive reactive oxygen species (ROS) production, which results in vascular smooth muscle cell (VSMC) senescence [11]. It has been revealed that an autophagy regulatory mechanism is mediated by EIF5A at the translational level, which can be harnessed to reverse immune senescence in humans [12]. Evidence from a rat model has shown that a reduction in EIF5A content is associated with brain aging [13].

EIF5A2, a second isoform of EIF5A, is upregulated in different types of tumor cells and is an important regulator of tumorigenesis in vitro [7, 14]. To further reveal the functions of EIF5A2 in vivo, Chen et al. generated EIF5A2 overexpressing transgenic mice. Unexpectedly, the EIF5A2 transgenic mice exhibited accelerated organismal aging phenotypes instead of forming spontaneous tumors. However, the mechanisms underlying the effects of EIF5A2 on aging remain to be further studied.

The purpose of this study was to address the effects of EIF5A2 on the transcription of aging-related genes. To this end, we established an EIF5A2 overexpression model in vitro and analyzed the impact of EIF5A2 on the level of gene expression by RNA-sequencing and analyzing the transcriptomes of the overexpressing cells compared to controls. Cell viability, proportion of senescent cells and the cell cycle were performed to evaluate the cell senescence. EIF5A2-regulated transcription factor (TF) and target gene network analyses were also conducted. We then generated a transcription factor–target gene regulatory interaction network and verified the EIF5A2- tanscription factors (TFs)-target regulation network in microglia from human brain tissue via the existing database. Our results should expand the current understanding of EIF5A2-mediated aging regulation.

## Material and methods

### EIF5A2 cloning and plasmid construction

The primer pairs for Hot Fusion were designed with CE Design V1.04, in conjunction with the portion of the vector pIRES-hrGFP-1a sequences, each with a 17 bp–30 bp overlap.

- F-primer: agcccgggcggatccgaattcATGGCAGACGAAATTGATTTCAC
- R-primer: gtcatccttgtagtcctcgagTTTGCAGGGTTTTATGGCTACA

Vector pIRES-hrGFP-1a was digested by EcoRI and XhoI (NEB, Ipswich, MA, US) at 37℃ for 2 h. The enzyme-digested vector was then run on a 1.0% agarose gel and purified with a Qiagen column kit (Qiagen, Dusseldorf, Germany). The total RNA was isolated from SH-SY5Y cells with Trizol. Purified RNA was transcribed to cDNA using an oligo dT primer. Then, the insert fragment was synthesized with polymerase chain reaction (PCR) amplification. A linearized vector digested by *EcoRI* and *XhoI* (NEB, Ipswich, MA, US) and PCR insert were added to a PCR microtube for ligation with ClonExpress® II One Step Cloning Kit (Vazyme, Nanjing, China). Plasmids were introduced into an *Escherichia coli* strain by chemical transformation. Cells were plated onto LB agar plates containing 1 µL/mL ampicillin and incubated overnight at 37℃. Colonies were screened by colony PCR (28 cycles) with universal primers (located on the backbone vector). The insert sequence was verified by Sanger sequencing.

### Cell culture and transfection

The human neuroblastoma cells (SH-SY5Y cell line; Procell Life Science & Technology Co., Ltd., Wuhan, China) were cultured in Dulbecco’s Modified Eagle’s Medium /F12 medium (Sigma Aldrich, St. Louis, MO, USA) supplemented with 10% fetal bovine serum (FBS; Sigma Aldrich), 100 IU/mL penicillin (Sigma Aldrich), 100 µg/mL streptomycin (Sigma Aldrich), and glutamine (2 mM) (Sigma Aldrich) for 24 h. The transfection of SH-SY5Y cells with an EIF5A2-overexpressing (OE) plasmid was performed using Lipofectamine 2000 (Invitrogen, Carlsbad, CA, USA) according to the manufacturer’s instructions. The experiments were carried out at 48 h after transfection.

### Assessment of EIF5A2 overexpression

Glyceraldehyde -3-phosphate dehydrogenase (GAPDH) was used as the control gene for assessing the effect of EIF5A2-OE. The control group referred to the SHSY5Y transfected with empty vector. cDNA synthesis was performed according to standard procedures and RT-qPCR was carried out on the Bio-Rad S1000 with Bestar SYBR Green RT-PCR Master Mix (DBI Bioscience, Shanghai, China). The information of the primers used in the RT-PCT is presented in Additional File 1. The concentration of each transcript was normalized to the mRNA level of GAPDH using the 2^−ΔΔCT^ method [15]. Comparisons were performed using the paired Student’s *t*-test using GraphPad Prism software (San Diego, CA, USA).

### Western blot analysis

Proteins from normal and EIF5A2-OE SH-SY5Y cells were separated by electrophoresis on 10% sodium dodecyl sulfate polyacrylamide (SDS-PAGE) gel and subsequently transferred onto a polyvinylidene fluoride (PVDF) membrane (Millipore, San Jose, CA, USA). The blots were cut prior to hybridisation with antibodies. The overexpression of EIF5A2 was detected using a monoclonal Flag antibody (Sigma-Aldrich) diluted in tris-buffered saline tween (TBST) (1:2000) and GAPDH was used as the loading control.

### RNA extraction for library preparation and sequencing

Total RNA was extracted with TRIZOL (Life Technology, Carlsbad, CA, USA) as previous study descripted [16]. The RNA was further purified with two phenol–chloroform treatments and then treated with RQ1 DNase (Promega, Madison, WI, USA) to remove DNA. The quality and quantity of the purified RNA were redetermined by measuring the absorbance at 260 nm/280 nm (A260/A280) using Smartspec Plus (BioRad, Hercules, CA, USA). The integrity of RNA was further verified by 1.5% agarose gel electrophoresis. For each sample, 1 μg of the total RNA was used for RNA-seq library preparation by VAHTS Stranded mRNA-seq Library Prep Kit (Vazyme, Nanjing, China). Polyadenylated mRNAs were purified and fragmented, and then converted into double strand cDNA. After the end repair and A tailing step, the DNAs were ligated to VAHTS RNA Adapters (Vazyme, Nanjing, China). Purified ligation products corresponding to 200‒500 bps were digested with heat-labile UDG, and the single strand cDNA was amplified, purified, quantified, and stored at − 80ºC before sequencing. For high-throughput sequencing, the libraries were prepared following the manufacturer’s instructions and applied to an Illumina HiSeq X Ten system for 150 nt paired-end sequencing.

### RNA-seq raw data clean and alignment

Raw reads containing more than 2-N bases were first discarded. Then, adaptors and low-quality bases were trimmed from raw sequencing reads using FASTX-Toolkit (Version 0.0.13). The short reads less than 16 nt were also dropped. After that, clean reads were aligned to the GRch38 genome by tophat2 allowing 4 mismatches. Uniquely mapped reads were used for gene read number counting and fragments per kilobase of transcript per million fragments mapped (FPKM) calculation as previous study descripted [16].

### Differentially Expressed Genes (DEGs) analysis

The R Bioconductor package edgeR was utilized to screen out the differentially expressed genes (DEGs). A false discovery rate < 0.05 and fold change > 2 or < 0.5 were set as the cut-off criteria for identifying DEGs as previous study descripted [16].

### Real-time qPCR validation of DEGs

To further validate the RNA-seq data, RT-qPCR was performed for DEGs. The primer information used for RT-PCT is presented in Additional File 1. Total RNA remaining from the RNA-seq library preparation was used for RT-qPCR. RNA was reverse transcribed into cDNA using a M-MLV Reverse Transcriptase (Vazyme). RT-qPCR was performed with the StepOne RealTime PCR System using the SYBR Green PCR Reagents Kit (Yeasen, Shanghai, China). The PCR conditions consisted of denaturation at 95 °C for 10 min, 40 cycles of denaturation at 95 °C for 15 s, annealing and extension at 60 °C for 1 min. PCR amplifications were performed in triplicate for each sample. The RNA expression levels of all genes were normalized against GAPDH.

### Functional enrichment analysis

To sort out functional categories of DEGs, Gene Ontology (GO) terms and Kyoto Encyclopedia of Genes and Genomes (KEGG) pathways were identified using KOBAS 2.0 server [17]. The hypergeometric test and Benjamini–Hochberg false-discovery rate (FDR) controlling procedure were used to define the enrichment of each term (FDR < 0.05).

### CCK-8 assay

The viability of SH-SY5Y cells was measured by a Cell Counting Kit-8 (Dojindo Laboratories, Shanghai, China) on the basis of the manufacturer’s instructions and previous research [18]. Briefly, after being inoculated in 96-well plates (2 × 10^4^ cells/well), the control group and the SH-SY5Y cells transfected with EIF5A2-OE plasmid were incubated in a humidified incubator at 37 ℃ with 5% CO_2_ for 0 h, 24 h, 48 h and 72 h. Then the cells were supplemented with 20 μL of the CCK-8 solution and incubated for 4 h. The absorbance at 450 nm was measured using a microplate reader (PerkinElmer/Envision, PerkinElmer, Inc. USA).

### Senescence-associated β-galactosidase (SA-β-gal) staining

A Senescence β-Galactosidase Staining Kit (Beyotime, Shanghai, China) was used to evaluate the senescence status of SH-SY5Y cells according to the manufacturer's instructions and previous research [18]. Briefly, cells were washed with phosphate-buffered saline (PBS, Life Technologies, Shanghai, China) and fixed in SA-β-gal fixing solution for 15 min at room temperature. Subsequently, cells were washed three times (3 min/time) with PBS and stained with SA-β-gal working solution at 37 °C overnight. The β-galactosidase positive cells were calculated using ImageJ software (Version 1.52v, National Institutes of Health, Bethesda, MD) while its status was observed under a microscope (BX53M, OLYMPUS, Tokyo, Japan).

### Cell cycle assay

The SH-SY5Y cells were fixed with precooled 70% ethanol at 4˚C overnight. After being re‑suspended in PBS and cultured with RNAase (10 mg/ml) at 4˚C for 1 h, the cells were incubated with propidium iodide (PI; 10 µg/ml) solution in the dark for 15 min. Cell cycle was evaluated with the FACSCalibur Flow Cytometer (BD Biosciences; Becton, Dickinson and Company) as previous research reported [18]. The data were processed by CellQuest software (version 5.1; BD Biosciences; Becton, Dickinson and Company).

### Analysis of EIF5A2-regulated TFs and target gene networks in glial cells

In order to obtain the target genes of differentially expressed TFs, we gathered candidate genes from identified DEGs from our differential analysis. HOMER, a software suite for ChIP-Seq analysis, was adopted to identify the enriched motifs from the promoter region of the DEGs [19]. Known TFs and corresponding transcription factor binding sites were got from JASPAR databases [20]. Then, to further decipher the TF-motif regulatory relationships we predicted the targeted motifs of TFs by searching the binding sites of TFs within the promoter region of candidate target genes using TargetScan (release 7.2: March 2018). After overlapping the enriched motifs selected by HOMER and the motifs bound to the known TFs which were found with TargetScan, EIF5A2-TFs-targets regulation networks were constructed. Cytoscape (version 3.5.1) was used to visualize our regulation network. CentiScaPe app, a plug-in of Cytoscape, was used to calculate the degree distribution of the network.

### Downloading RNA-seq data of microglia from human brain tissue

The RNA-seq data of microglia from human brain samples were downloaded from the TCGA database (Data source: GSE99074) to analyze the expression of EIF5A2-TFs-targets [21].

## Results

### EIF5A2 overexpression in SH-SY5Y cells preferentially regulates the expression of several genes

**Fig. 1:**
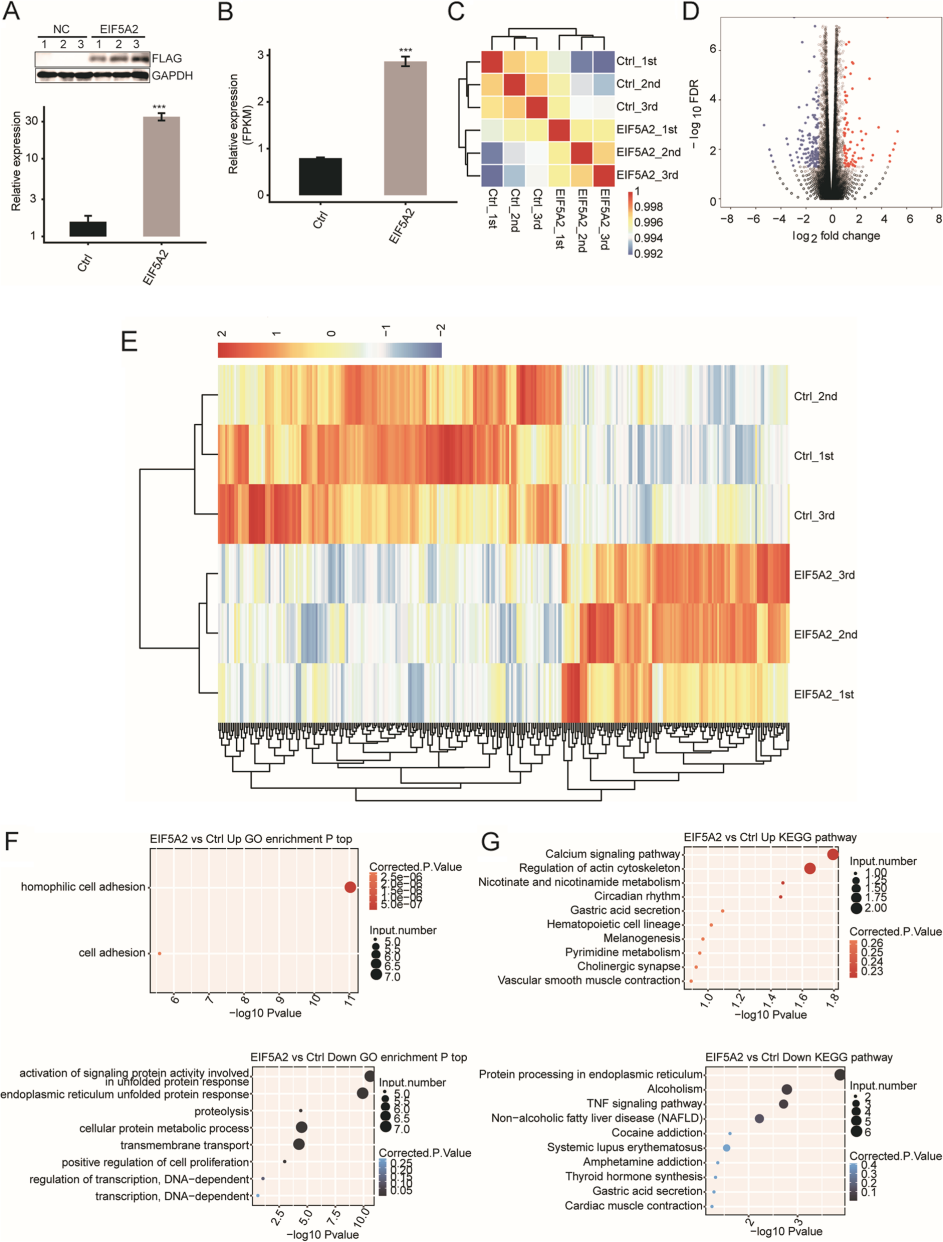
RNA-seq analysis of EIF5A2-regulated transcriptome. **A** EIF5A2 expression quantified by western blot (top panel) and RT-qPCR (bottom panel). (*n* = 3, ***p < 0.001) **B** EIF5A2 expression quantified by RNA-seq data. (*n* = 3, ***p < 0.001) **C** Heat map shows the hierarchically clustered Pearson correlation matrix resulted from comparing the transcript expression values for control and EIF5A2 overexpression samples. **D** Identification of EIF5A2 regulated genes. Up-regulated genes are labeled in red, whereas down-regulated are labeled in blue in the volcano plot. **E** Hierarchical clustering of DEGs in control and EIF5A2 overexpression samples. FPKM values are log2-transformed and then median-centered by each gene. **F** The representative GO biological processes of up-regulated and down-regulated genes. **G** The representative KEGG pathways of up-regulated and down-regulated genes

To reveal the EIF5A2-mediated transcriptional regulation, we performed RNA-seq experiments with EIF5A2-OE cells. Six cDNA libraries were prepared from the control and EIF5A2-OE cells (details can be found in Additional file 2). As shown in Fig. 1A and B, the efficacy of EIF5A2-OE was assessed by RT-qPCR, western blotting and FPKM calculation, respectively. The heat map showed the correlation coefficient matrix and three replicates of the EIF5A2-OE and the control group were highly correlated and the two groups were separated from each other (Fig. 1C. Details can be found in Additional file 3).

Three hundred sixteen DEGs were identified in total, with 190 downregulated genes (details can be found in Additional file 4) and 126 upregulated genes (details can be found in Additional file 5) respectively. The volcano plot was established to show the DEGs in EIF5A2-OE cells (Fig. 1D). The heat map analysis of the DEG expression patterns demonstrated that the consistency of the EIF5A2-mediated transcription in the replicate data sets was very high (Fig. 1E). All together, these results indicated that EIF5A2 extensively regulated gene expression.

Based on the cut-off criteria, the upregulated DEGs were enriched in 2 GO terms, and the downregulated DEGs were enriched in 8 GO terms (Fig. 1F). In the biological process terms of the GO analysis, the upregulated genes in the EIF5A2-OE cells were primarily associated with cell adhesion. Whereas the downregulated genes were closely related with the unfolded protein response (UPR) (Details of the down-regulated GO enrichment analysis can be found in Additional file 6). Some genes were dysregulated in the EIF5A2 overexpressing samples annotated with KEGG categories and were mainly involved in the calcium signaling pathway, and regulation of the actin cytoskeleton and protein process in the ER (Fig. 1G). As most of the pathways and cellular response were closely related to cell senescence, we assumed that the potential EIF5A2-regulated DEGs could play a substantial role in aging.

### EIF5A2 overexpression influences cell viability, cell cycle and cell senescence

**Fig. 2:**
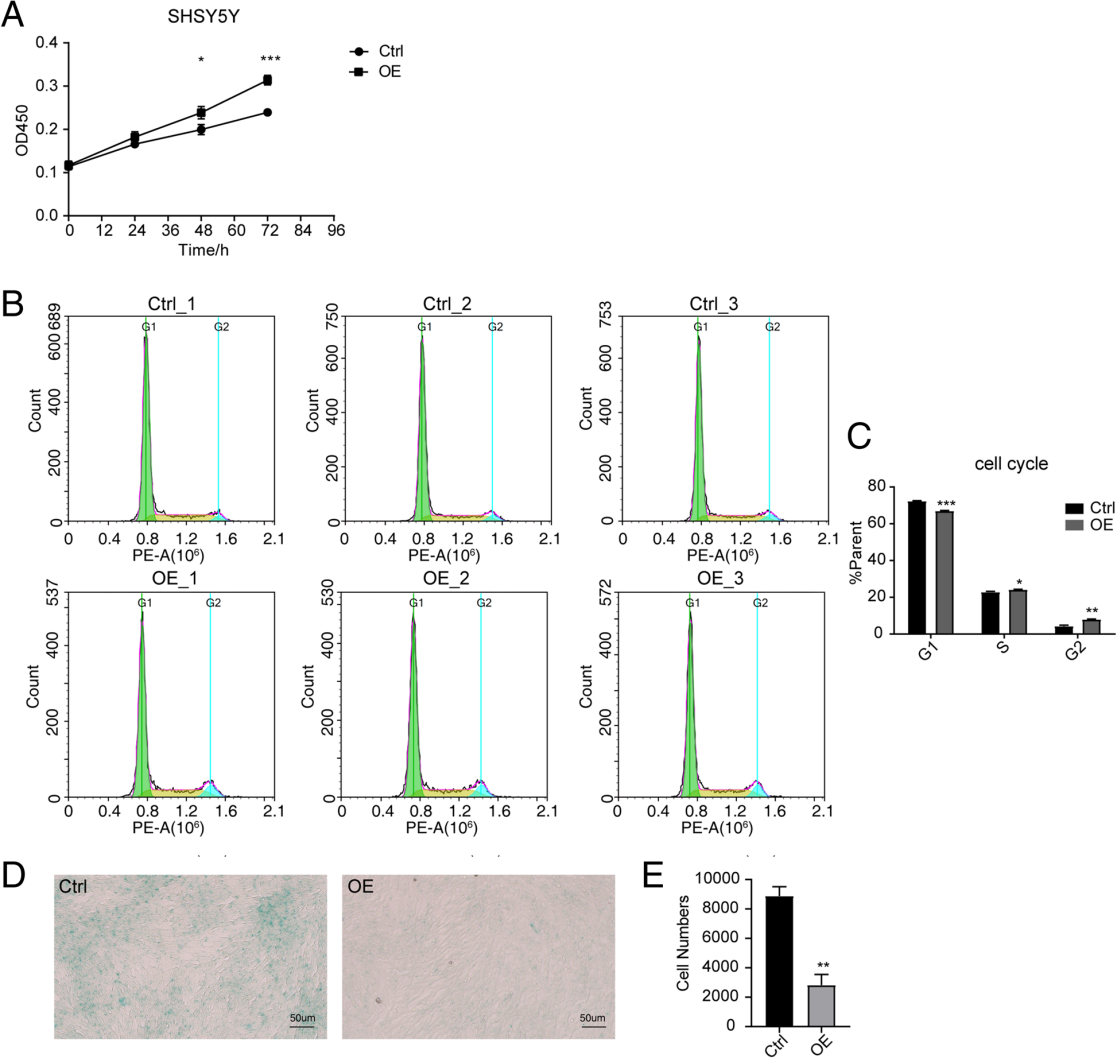
Cell senescence alleviates by EIF5A2 overexpression. **A** SH-SY5Y cells were transfected with the control vector or EIF5A2 overexpression plasmids. CCK8 assay was performed to examine cell viability at 0, 24, 48, and 72 h. **B**-**C** Cell-cycle progression in SH-SY5Y cells was evaluated with flow cytometry 48 h after transfection with EIF5A2 overexpression and the control plasmids. **D**-**E** The senescence of SH-SY5Y cells was identified by the cell senescence-associated β-galactosidase staining. (*n* = 3, *p < 0.05, **p < 0.01, ***p < 0.001.)

To evaluate the effect of EIF5A2-OE on cell senescence, the cell viability, senescence and cycle of the SH-SY5Y cells were determined by CCK-8, SA-β-gal staining and flow cytometry, respectively. After being treated EIF5A2 overexpression plasmid at different time intervals (0, 24, 48 and 72 h), cells showed enhanced viability in a time-dependent manner (*p < 0.05, ***p < 0.001; Fig. 2A). Moreover, the proportion of SH-SY5Y cells was down-regulated in G1 phase but up-regulated in S and G2 phases compared with control group (*p < 0.05, **p < 0.01, ***p < 0.001; Fig. 2B-C). As shown in Fig. 2D-E, the blue cytoplasm indicated SA-β-gal staining positive cells. The proportion of the positive cells significantly decreased in the EIF5A2-OE group (**p < 0.01), suggesting SH-SY5Y cell senescence was reduced by overexpressed EIF5A2 in cells. These results suggested that EIF5A2-OE promoted cell viability, reduced cell cycle arrest in G1 phase and alleviated cell senescence.

### EIF5A2 selectively regulates the expression of UPR associated genes

**Fig. 3:**
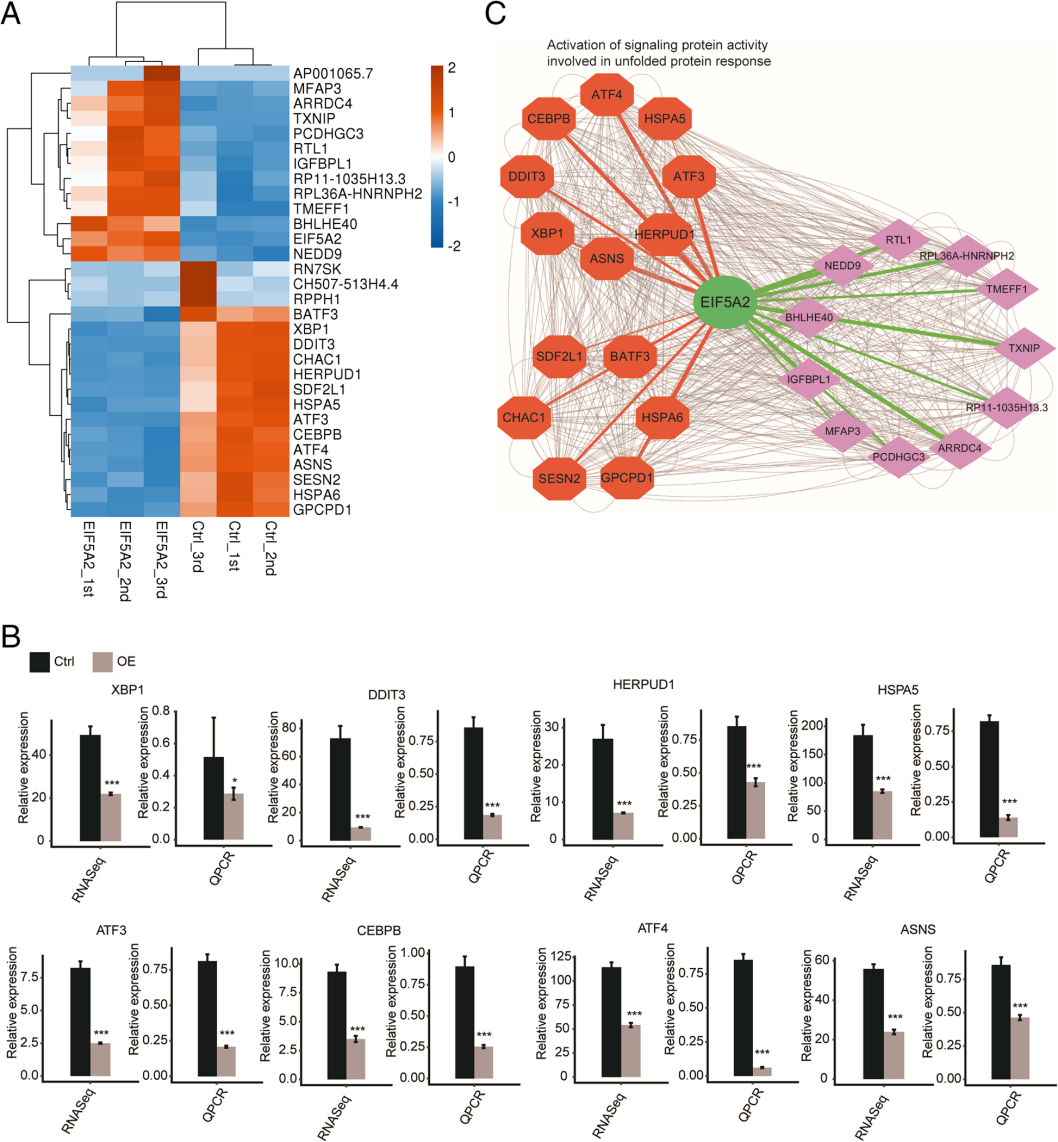
EIF5A2 selectively regulates the expression of aging associated genes. **A** Hierarchical clustering of the levels of 30 genes with the most significant expression differences. Expression values (FPKM) are log2-transformed and then median-centered by each gene. The genes in red were enriched in the GO biological process of activation of signaling protein activity involved in unfolded protein response. **B** Validation of the expression level of EIF5A2 regulated unfolded protein response (UPR) genes. Gene expression quantified by RNA sequencing data and RT-qPCR. (*n* = 3, ***p < 0.001) **C** The co-expression network of EIF5A2 and the UPR-related genes. The thickness of the line represents the correlation coefficient (correlation > 0.7, *p* < 0.01) between EIF5A2 and UPR-related genes. The color of the line represents a positive (green) or negative(red) correlation. The grey lines represent correlation relationship among the UPR-related genes

To further investigate the underlying mechanisms of EIF5A2 on cell aging, 30 genes with the most significant expression differences were identified from all the DEGs regulated by EIF5A2 overexpression (Fig. 3A). Among these 30 genes, eight genes were enriched in the UPR according to the GO biological process. These eight genes were *ASNS*, *ATF3*, *ATF4*, *CEBPB*, *DDIT3*, *HERPUD1*, *HSPA5*, and *XBP1*(Fig. 3A The 8 genes were marked in red.), confirming again that EIF5A2 overexpression has a significant impact on the UPR. The expression levels of these eight genes were further verified with RNA-seq experiments and RT-qPCR (Fig. 3B. Details of the PCR primer pairs used here can be found in Additional file 1). The expression of these eight genes was markedly reduced in the EIF5A2-OE group, strongly suggesting that EIF5A2 might manipulate the UPR via these genes.

The correlations between the expression of these 30 genes and the level of EIF5A2 were analyzed, and the hierarchical clustering mapping was performed according to the magnitude of the correlation (Fig. 3C). Correlation analyses indicated that the levels of these eight UPR-related genes were significantly negatively associated with EIF5A2 expression (details of the correlations of UPR-related genes and EIF5A2 used here can be found in Additional file 7). The exact correlation coefficients were as follows: *ASNS* (*r* =  − 0.99, *P* < 0.001), *ATF3* (*r* =  − 0.98, *P* < 0.001), *ATF4* (*r* =  − 0.98, *P* < 0.001), *CEBPB* (*r* =  − 0.98, *P* < 0.001), *DDIT3* (*r* =  − 0.95, *P* < 0.01), *HERPUD1* (*r* =  − 0.95, *P* < 0.01), *HSPA5* (*r* =  − 0.92, *P* = 0.01), and *XBP1* (*r* =  − 0.94, *P* < 0.01). As the relationship between abnormal protein folding and aging diseases is widely known, these results confirmed that EIF5A2 might influence the aging process via regulation of the UPR.

### EIF5A2-regulated TFs and target gene networks in SH-SY5Y cells

**Fig. 4:**
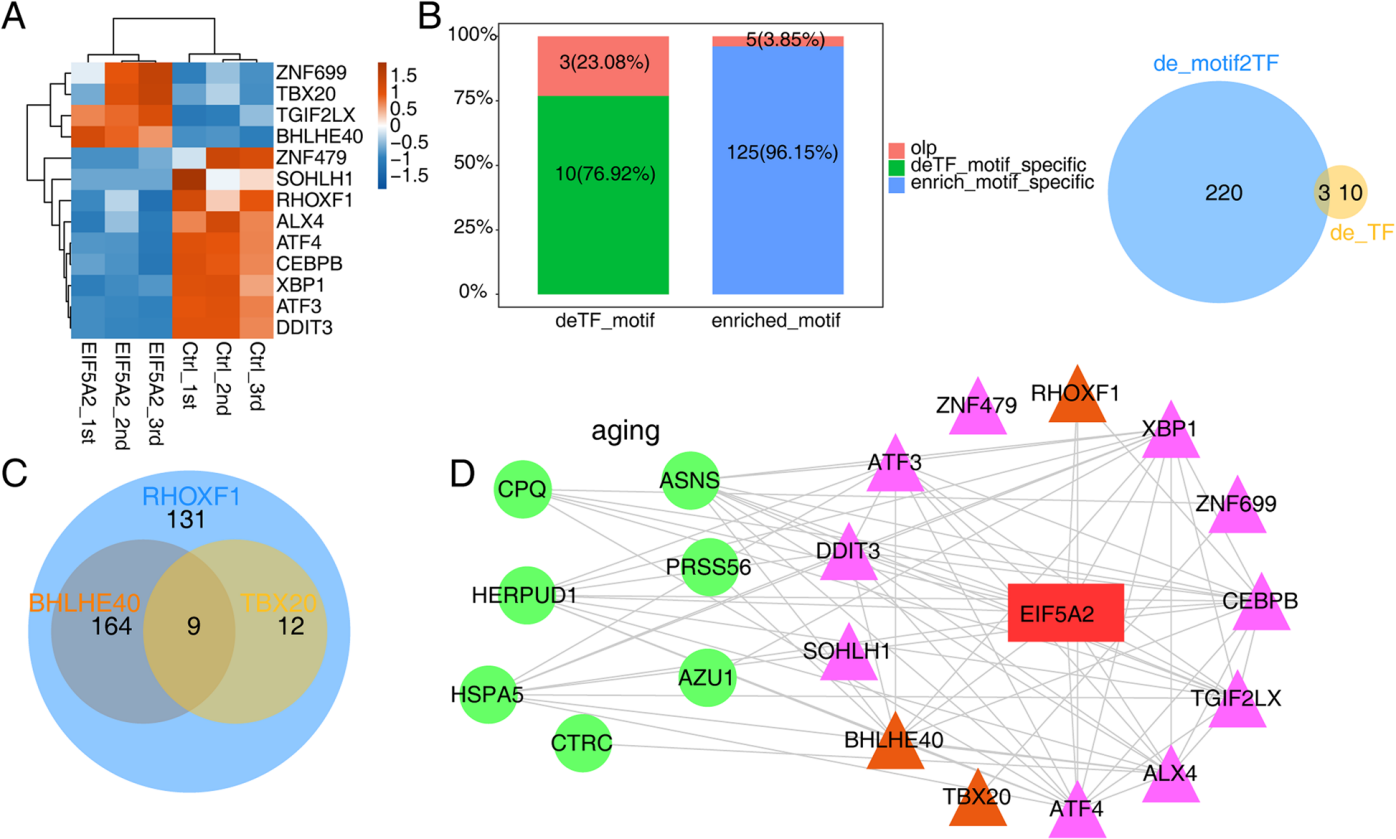
EIF5A2-regulated transcription factors and target gene networks in SH-SY5Y cells. **A** Hierarchical clustering of differentially expressed transcription factors regulated by EIF5A2. The expression values of each gene were normalized by row. **B** Bar plot showing the overlap and specific number of EIF5A2-regulated TF target motifs (deTF_motif) and motifs enriched in promoters (enriched_motif) of all DEGs (left panel). Venn diagram showing the overlap of EIF5A2-regulated TFs (de_TF) and all potential TFs (de_motif2TF) that may regulate the DEGs (right panel). **C** Venn diagram of the DEG targets with potential binding motifs of the three EIF5A2-regulated transcription factors in the promoter region. **D** EIF5A2-TFs-targets regulation network in SH-SY5Y cells. Triangles represent transcription factors. Spots represent DEGs, excluding TFs. Aging associated DEGs were in green. EIF5A2-regulated TFs which may targeting the DEGs identified from RNA-seq data are marked in orange. Pearson product-moment correlation coefficient > 0.9 or < -0.9, *p*-value < 0.05

The above findings illustrated that EIF5A2 played a role in regulating the expression of UPR associated genes among which DNA-binding TFs were enriched as well. After screening the TF genes among DEGs following overexpression of EIF5A2, it was found that there were significant changes in the levels of 13 TFs (Fig. 4A. Details can be found in Additional file 8). Thus, we hypothesized that EIF5A2 might exert its effects of transcription regulation of UPR by regulating the expression of certain TFs. To verify this hypothesis, we generated a TF–target gene regulatory interaction network as previous study descripted [22]. Firstly, in order to predict the TFs whose motifs were enriched in the promoter regions of the DEGs unbiasedly, we selected the sequence in the promoters of all the DEGs, and ran HOMER for the known motif search. We performed four independent analyses with different sets of promoter regions: 1 kb, 2 kb, 3 kb upstream and downstream of the transcriptional start site, and 5 kb upstream of the transcriptional start site. We chose the motifs enriched in at least two groups of the promoter sets, resulting in a total of 130 motifs. Five out of these enriched motifs were overlapped with three motifs of EIF5A2-regulated TFs, corresponding to 13 TFs (Fig. 4B left panel). Next, we constructed the association between differentially expressed TFs and all potential TFs that might regulate the DEGs in EIF5A2-OE cells, three TFs were finally identified as regulatory TFs: BHLHE40, RHOXF1 and TBX20 (Fig. 4B. right panel). The *P*-values for the three TFs were as followed: BHLHE40 (*P* < 0.01), RHOXF1 (*P* < 0.01) and TBX20 (*P* < 0.001) (Details can be found in Additional file 9). These results supported the hypothesis that EIF5A2-regulated TFs are involved in the regulation network of EIF5A2.

We then overlapped the target genes of BHLHE40, RHOXF1 and TBX20, we found 9 genes that were under mutual regulation of these three TFs. Interestingly, the eight genes (*ASNS*, *ATF3*, *ATF4*, *CEBPB*, *DDIT3*, *HERPUD1*, *HSPA5*, and *XBP1*) that associated with UPR and verified in Fig. 3 were among these 9 target genes (Fig. 4C. Details can be found in Additional file 9). The associations between the eight genes and the three TFs were showed in Fig. 4D. The details of the correlations between these three differentially expressed TFs and the validated UPR-related genes in Fig. 4D can be found in Additional file 10.

In summary, these results demonstrated that overexpression of EIF5A2 led to the differential expression of three TFs, including BHLHE40, RHOXF1 and TBX20. Therefore, we assumed that EIF5A2 might regulate the expression of eight UPR-related genes through these three TFs, thus might be associated with aging process.

### EIF5A2-TFs-targets regulation network in microglia from human brain tissue

**Fig. 5:**
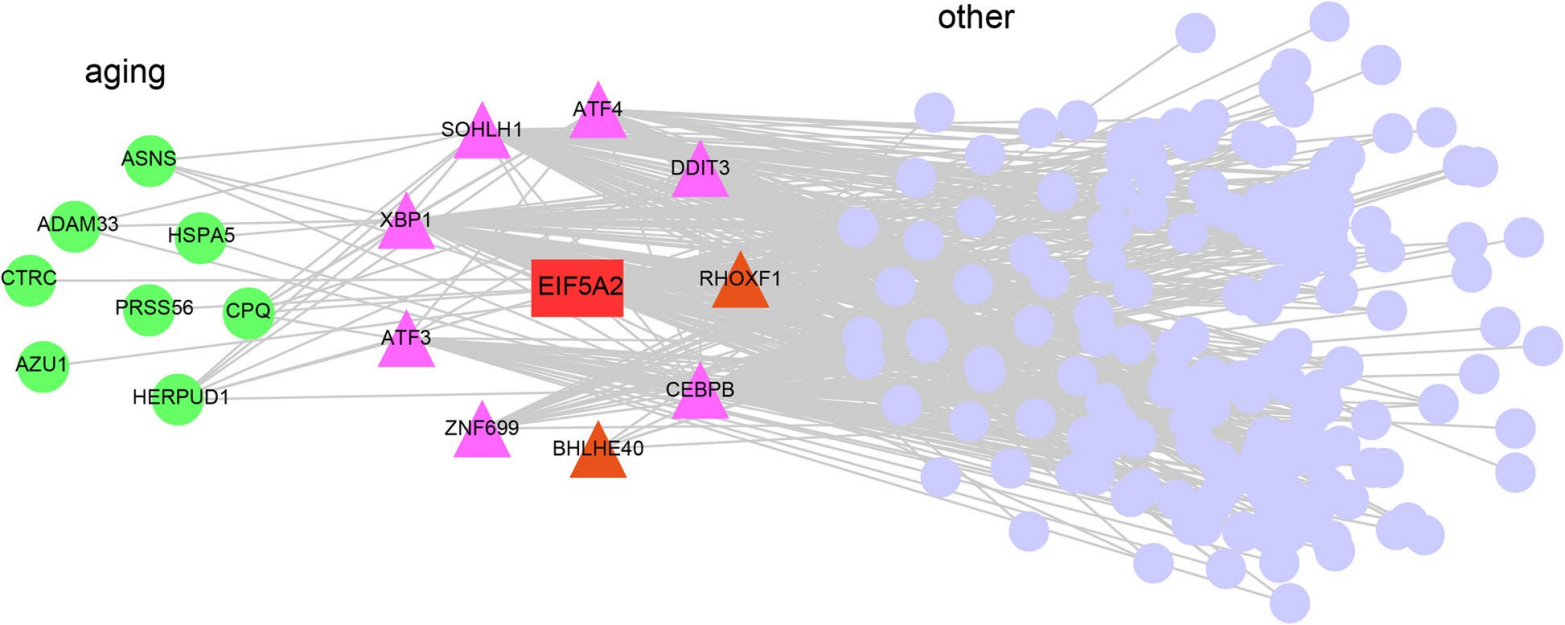
EIF5A2-TFs-targets regulation network in microglia from human brain tissue. Triangles represent transcription factors. Spots represent DEGs, excluding TFs. Aging associated DEGs were in green, while the others were in purple. EIF5A2-regulated TFs which may targeting the DEGs identified from RNA-seq data are marked in orange. Data source: GSE99074. Pearson product-moment correlation coefficient > 0.4 or < -0.4, *p*-value < 0.05

The results of the study above were further verified with the published database of human glial cell tissue (Fig. 5). With the database Galatro TF et al. not only presented an extensive collection of human microglial gene expression profile, but also illustrated the age-related changes occurred [21]. In this case, the database was an appropriate option for the verification of our above finding. However, considering the large numbers of genes contained in the database, we could not but choose 0.4 as the correlation threshold which consequently achieve just a medium correlation. To make the results more reliable, we additionally set the *P*-value < 0.05 for the analyses. It was found that EIF5A2 was positively correlated with BHLHE40 and *ASNS* expression, but negatively correlated with *CEBPB*, *HERPUD1*, and *XBP1*. Both EIF5A2 and RHOXF1 were correlated to *NEDD9* and *ULK4P2* (details can be found in Additional file 11). Meanwhile *HERPUD1* was significantly correlated with *ATF3*, *ATF4*, and *DDIT3*. *CEBPB* was significantly correlated with *HSPA5* (details can be found in Additional file 11). However, unlike our finding in vitro*, TBX20* showed no relation with EIF5A2 in this published database of human glial cell tissue. It was worth noting that there were still some limitations of the above results concluded from the published database, such as the potential inconsistency of the study design and the variability of the data quality. Further clinical studies on the EIF5A2-TFs axis in age-related genes and diseases need to be explored.

## Discussion

In this study, we profiled the entire transcriptome in a human neuroblastoma cell line (SH-SY5Y cells) following the overexpression of EIF5A2, which allows for the decoding of the EIF5A2-mediated regulation of gene expression. Interestingly, upon EIF5A2 overexpression, the expression of genes associated with the UPR was specifically downregulated. Moreover, EIF5A2 preferentially regulated the DEGs enriched in the negative regulation of UPR-related genes as follows: *ASNS*, *ATF3*, *ATF4*, *CEBPB*, *DDIT3*, *HERPUD1*, *HSPA5*, and *XBP1*. By establishing the EIF5A2-TFs-targets correlation network with EIF5A2 overexpressing cells, we bioinformatically identified three TFs (BHLHE40, RHOXF1 and TBX20) that might target at the eight UPR-related genes with statistical significance. Considering the results above we speculated that EIF5A2 might modulate the aging process via regulation of TF–target genes associated with UPR.

Cell aging refers to a cell state implicated in a wide spectrum of physiological processes and could be targeted to slow the aging process [23, 24]. Unfolded protein response, proteolytic processing by proteases, cell proliferation and transmembrane transport were all profoundly modulated the cellular senescence and thus age-related diseases [25–28]. As the high-throughput technologies developed so rapidly, we can explore senescent cells at the single-cell level and potentially uncover new gene targets. In our present study, we subjected all 316 DEGs to GO and KEGG annotation. The representative GO biological processes of down-regulated genes were enriched in the terms of “unfolded protein response”, followed by “proteolysis” and “transmembrane transport”. While DEGs enriched in “regulation of cell proliferation” and “DNA-dependent transcription” were unexpectedly much less. In Additional file 6 we itemized the DEGs and *P* value in each biological process identified in the GO annotation.

The unfolded or misfolded proteins that accumulate inside the endoplasmic reticulum (ER) lumen might result in many aging-related diseases. The accumulation of these proteins would increase ER stress and activate UPR which in turn could protect cells from stress and facilitate the restoration of cellular homeostasis [29, 30]. The UPR reduces the protein load by upregulating ER-chaperones, such as binding immunoglobulin protein (BiP), and by attenuating protein translation via eukaryotic initiation factor 2 alpha (eIF2alpha) phosphorylation [30]. The regulatory role of the UPR in senescent cells has been under abundant scrutiny. Studies have shown that the UPR in senescent cells differed markedly from younger cells in the aspect of the level of cell apoptosis and chaperone activity [31]. Chadwick et al. recently used chronologically aged yeast cells to illuminate how the UPR activated upon accumulation of misfolded proteins affected longevity [32]. They found a significant increase in the misfolded protein burden in the ER during the process of aging, which consequently augmented UPR activation. Our study found that the expression of several UPR-related gene and TFs was significantly reduced in cells when EIF5A2 was overexpressed. Thus, we assumed that EIF5A2 might influence the aging process by manipulating the UPR.

To further investigate the mechanisms underlying the effects of EIF5A2 on the UPR, we identified 30 genes with the most significant expression differences, from which 8 genes were enriched in the activation of signaling protein activity involved in the UPR according to the GO biological process. These 8 genes were *ASNS*, *ATF3*, *ATF4*, *CEBPB*, *DDIT3*, *HERPUD1*, *HSPA5*, and *XBP1*. *ATF4*, a DNA-binding factor that modulates responses to amino acid availability and ribosomal function, has been shown to play an important role in mediating the aging of mice [33]. It has been proven that resveratrol, one of the most well-known drugs used in the treatment of aging, exerts its anti-aging biological functions mainly through protein heterodimerization activity, cytokine receptor binding, phosphatase binding, and the protein phosphatase binding process [34]. Further animal experimental validation with *ATF4*(+ / −) (knockdown) in naturally aging mice illustrated that resveratrol substantially inhibits intestinal aging via downregulation of the *ATF4*/Chop/Bcl-2/Bax signaling pathway [35]. Consistent with this research, our study identified *ATF4* as an important DEG regulated by EIF5A2-OE. The amino acid deprivation-activation kinase GCN2 participates in the mediation of amino acid availability, which could attenuate mRNA translation in cells and help to extend the lifespan in model organisms [36]. *ATF4*, the downstream transcription factor of GCN2, plays a crucial role in mediating 4E-BP induction. By binding to *ATF4*-binding sites of the 4E-BP intron, *ATF4* regulates 4E-BP transcription during normal development, then influences the process of aging.

*ATF3* plays a pleiotropic role in biological processes through genotoxic stress. Using *ATF3* knockdown and overexpression, Kim et al. found that *ATF3* contributes to acrylamide-induced senescence by enhancing ROS production, activating p38 and JNK kinases, and promoting the *ATF3*-dependent expression of p53, which results in regulation of cellular senescence in macrophages [37]. Correlation analyses of our research illustrated that the levels of *ATF4* and *ATF3* were significantly negatively associated with EIF5A2 expression (*P* < 0.001), indicating that EIF5A2 might affect aging through *ATF4* and *ATF3*. However, the specific effects and mechanism of EIF5A2 remain to be further investigated.

When the effect of LncRNA-ES3 was studied in high glucose-induced senescence of vascular smooth muscle cells (HA-VSMCs), researchers found that the expression of BHLHE40 was decreased significantly in HA-VSMCs, and overexpression of BHLHE40 alleviated senescence of HA-VSMCs [38]. While some studies reported that hypothyroidism might contribute to extended lifespan of humans, a recent study indicated that BHLHE40 was responsive to triiodothyronine (T3), suggesting that BHLHE40 could be a novel target gene for the thyroid hormone receptor and play an important role in cell aging [39]. Our research has shown that BHLHE40 was significantly up-regulated in EIF5A2-OE cells, thus we assumed that BHLHE40 might be a potential target by which EIF5A2 influenced aging. Notably, because cellular aging differs across cell types and in terms of dysregulated genes, these potential EIF5A2-regulated DEGs could be complicated by the contribution of other factors.

In this study, we have successfully applied RNA-seq technology to demonstrate the potential effects of EIF5A2 on aging. We demonstrated that eight genes closely related to the UPR were downregulated in EIF5A2-OE human neuroblastoma cells (SH-SY5Y cell line). Moreover, using the EIF5A2-TFs-targets regulation network and further verified via the published database of human glial cell tissue, we found TFs BHLHE40 and RHOXF1 through which EIF5A2 modulated the eight target genes associated with UPR. Therefore, we assume that EIF5A2 represents a novel target for managing aging. However, functional studies will be required to confirm the biological relevance of EIF5A2 on aging in vivo.

## Supplementary Information

**Additional file 1. **Primers sequence. Information of primers used in RT-qPCR experiments throughout the present study.

**Additional file 2.** RNA-seq reads of SH-SY5Y cells from control group and EIF5A2-OE group.

**Additional file 3. **The correlation matrix of three biological replicates of the EIF5A2-OE group and the control group.

**Additional file 4. **The downregulated DEGs of EIF5A2-OE group vs Control group.

**Additional file 5. **The upregulated DEGs of EIF5A2-OE group vs Control group.

**Additional file 6. **Representative GO biological processes of down-regulated DEGs.

**Additional file 7. **Correlation of UPR-related genes and EIF5A2.

**Additional file 8. **Differentially expressed TFs in EIF5A2-OE group.

**Additional file 9. **Analysis of the potential target genes regulated by BHLHE40, RHOXF1, and TBX20.

**Additional file 10. **Correlation analysis between differentially expressed TFs and the validated UPR-related genes.

**Additional file 11. **Correlation analysis between DEGs and EIF5A2 in human glial cell tissue database.

**Additional file 12. **Gels and blots of Figure 1A.

## Data Availability

The data discussed in this publication are available under GEO Series accession number GSE159473.

## Abbreviations

EIF5A: Eukaryotic initiation factor 5A
VSMC: Vascular smooth muscle cell
TF: Transcription factor
PCR: Polymerase chain reaction
EIF5A2-OE: EIF5A2 overexpressing
GAPDH: Glyceraldehyde-3-phosphate dehydrogenase
FPKM: Fragments per kilobase of transcript per million fragments mapped
DEGs: Differentially expressed genes
GO: Gene Ontology
KEGG: Kyoto Encyclopedia of Genes and Genomes
SA-β-gal: Senescence-associated β-galactosidase
UPR: Unfolded protein response
