# Supplementary figures and images for: EIF5A2 specifically regulates the transcription of aging-related genes in human neuroblastoma cells
Source: BMC Geriatr. 2023 Feb 7;23:83. doi: 10.1186/s12877-023-03793-6 (PMC9906866; doi:10.1186/s12877-023-03793-6)

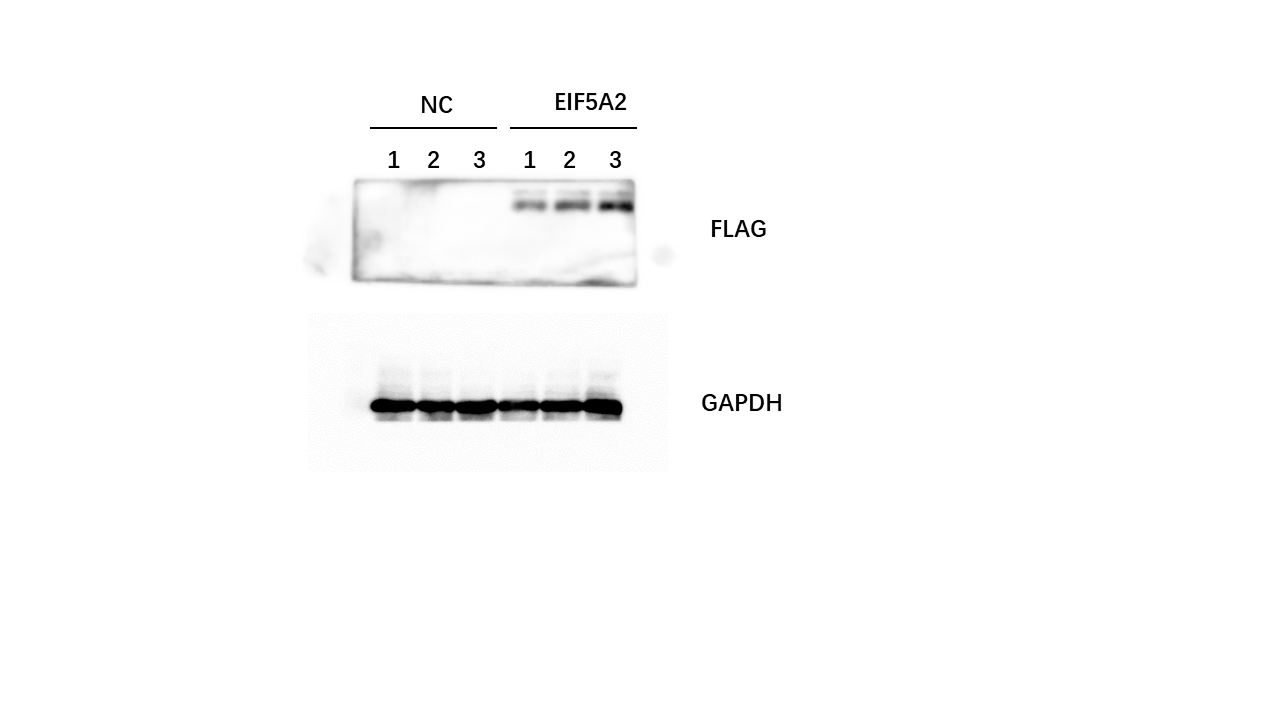

Supplement: Supplementary file 12 — Additional file 12. Gels and blots of Figure 1A. [file 12877_2023_3793_MOESM12_ESM.tif]
